# IL-6 Cytokine Family: A Putative Target for Breast Cancer Prevention and Treatment

**DOI:** 10.3390/ijms23031809

**Published:** 2022-02-05

**Authors:** Carla M. Felcher, Emilia S. Bogni, Edith C. Kordon

**Affiliations:** 1 Instituto de Fisiología, Biología Molecular y Neurociencias (IFIBYNE), Universidad de Buenos Aires—Consejo Nacional de Investigaciones Científicas y Técnicas (IFIBYNE-UBA-CONICET), Ciudad Autónoma de Buenos Aires (CABA) 1428, Argentina; carfelcher@gmail.com (C.M.F.); bogniemilia@gmail.com (E.S.B.); 2 Departamento de Química Biológica, Facultad de Ciencias Exactas y Naturales, Universidad de Buenos Aires, Ciudad Autónoma de Buenos Aires (CABA) 1428, Argentina

**Keywords:** breast cancer, IL-6, LIF, OSM, IL-11, mastitis, tumor microenvironment

## Abstract

The IL-6 cytokine family is a group of signaling molecules with wide expression and function across vertebrates. Each member of the family signals by binding to its specific receptor and at least one molecule of gp130, which is the common transmembrane receptor subunit for the whole group. Signal transduction upon stimulation of the receptor complex results in the activation of multiple downstream cascades, among which, in mammary cells, the JAK-STAT3 pathway plays a central role. In this review, we summarize the role of the IL-6 cytokine family—specifically IL-6 itself, LIF, OSM, and IL-11—as relevant players during breast cancer progression. We have compiled evidence indicating that this group of soluble factors may be used for early and more precise breast cancer diagnosis and to design targeted therapy to treat or even prevent metastasis development, particularly to the bone. Expression profiles and possible therapeutic use of their specific receptors in the different breast cancer subtypes are also described. In addition, participation of these cytokines in pathologies of the breast linked to lactation and involution of the gland, as post-partum breast cancer and mastitis, is discussed.

## 1. Introduction

The interleukin-6 (IL-6)/glycoprotein130 (gp130) cytokine family is a group of signaling molecules that are expressed widely and play multiple physiological roles across vertebrates [1,2]. They are involved in processes as diverse as hematopoiesis, inflammation, tissue remodeling, neuroprotection, cell differentiation, and cancer [3]. Each member of the family signals by binding to its specific receptor and at least one molecule of gp130, which is the common transmembrane receptor subunit for the whole group of cytokines [4]. In breast cancer, the most studied members of this family are interleukin-6 (IL-6), leukemia inhibitory factor (LIF), oncostatin M (OSM), and interleukin-11 (IL-11). In this review, we focus on the role of these cytokines in breast cancer development and their putative use as therapeutic targets.

**Figure 1:**
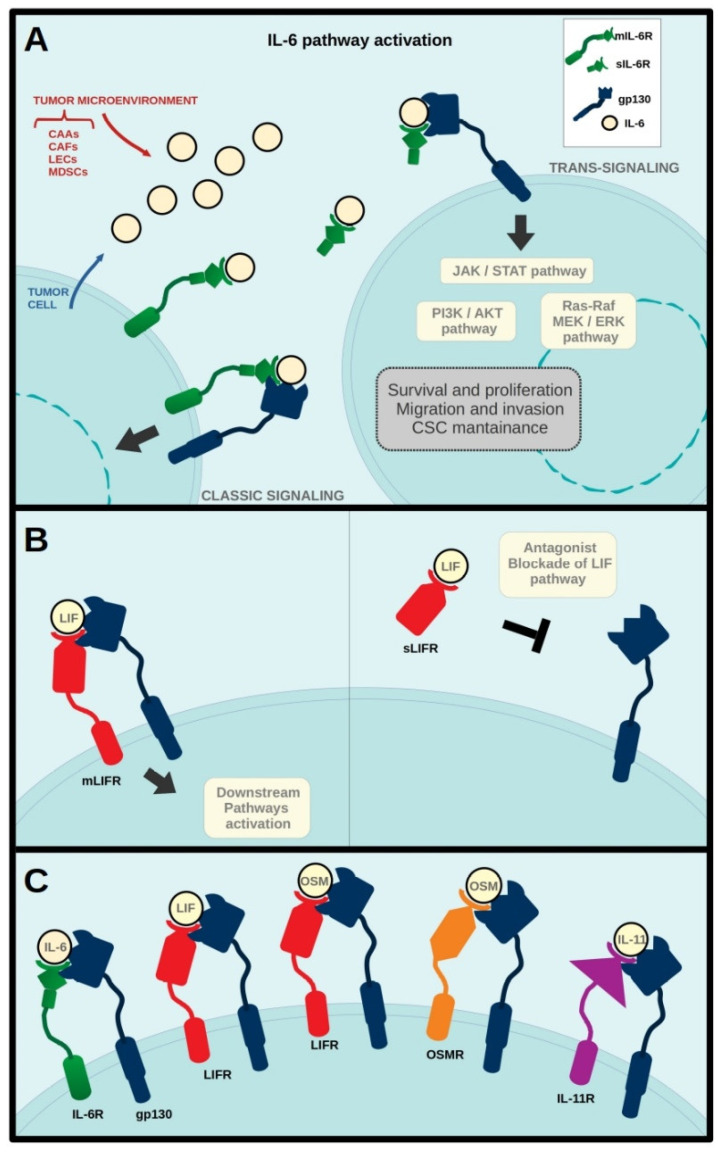
IL-6 cytokine pathway activation in breast cancer cells. IL-6 is secreted by tumor and stroma cells, such as cancer associated adipocytes (CAAs), cancer associated fibroblasts (CAFs), lymphatic endothelial cells (LECs), and myeloid derived stem cells (MDSCs). IL-6 binds to either transmembrane (mIL-6R) or soluble (sIL-6R) IL-6 receptor and to gp130 inducing ‘classic signaling’ through mIL-6R or ‘trans-signaling’ through sIL-6R (**A**). LIF binds to transmembrane LIF receptor (mLIFR) and gp130 inducing downstream signaling. LIF binding to soluble LIF receptors (sLIFR) blocks interaction with gp130 inhibiting LIF pathway activation (**B**). At the surface of target cells, secreted IL-6 cytokine family members bind to their specific receptors and gp130 to activate intracellular signaling cascades (**C**).

The first studies on the IL-6 cytokine family date back to the 1970s, when the importance of factors involved in B lymphocyte activation was highlighted for the first time. One of those soluble proteins, first cloned in 1986, was interleukin 6 (IL-6), which induced immunoglobulin production and therefore was initially named “B-cell stimulatory factor-2” [5]. It was later discovered that this molecule had been studied under many different names in multiple human and mouse tissues, where it showed pleiotropic functions [6,7,8]. IL-6 binds to the IL-6 receptor (IL-6R) and gp130 in a cooperative manner to form a competent signaling complex consisting of the hexamer [IL-6/IL-6R/gp130] × 2 [9]. Whereas gp130 is expressed in every human and mouse cell [10], IL-6R has only been detected in a few tissue types, which includes the mammary gland [11]. Only the binary complex of IL-6/IL-6R can interact with gp130 to form the signaling-competent complex, since gp130 alone has no measurable affinity for IL-6, and IL-6R alone does not bind gp130. It is worth noting that IL-6R exists not only as a transmembrane protein, but also as a soluble factor (sIL-6R), which also binds IL-6 with high affinity [12]. The complex formed by IL-6 and sIL-6R can interact with gp130 on cells that do not express IL-6R, thus triggering specific signaling events in cells which would be otherwise unresponsive to this cytokine [13]. This process is called trans-signaling and has been related to IL-6 pro-inflammatory effects, whereas the classic IL-6 signaling, via the membrane bound IL-6R, has been associated with regenerative or anti-inflammatory activities exerted by the cytokine [14] (Figure 1A).

LIF was first cloned in 1987 and was studied as an inducer of macrophage differentiation [15]. The murine and human LIF genes show 75% homology [16] and the mouse protein is known for maintaining self-renewal and pluripotency of embryonic stem cells, besides playing other relevant biological functions [17]. On target cell membranes LIF binds to a heterodimeric high-affinity complex composed by the LIF receptor β (LIFRβ) and gp130 [18]. LIF mRNA is subjected to alternative splicing, resulting in two isoforms that originate the transmembrane receptor (LIFR) and a secreted protein [19,20]. The soluble isoform of LIFR (sLIFR) binds LIF, but is unable to form the signaling competent complex with gp130. Then, sLIFR sequesters LIF in the extracellular microenvironment preventing its binding to LIFR and therefore inhibiting the intracellular signaling [21] (Figure 1B). On the other hand, the transmembrane complex LIFRβ-gp130 can also interact with secreted OSM, ciliary neurotrophic growth factor (CNTF), cardiotrophin 1 (CT1), and cardiotrophin-like cytokine (CLC) leading each of these factors to specific cell responses [22] (Figure 1C).

OSM was initially identified as a melanoma and other tumor cell growth inhibitor [23]. However, accumulated evidence now shows that this factor exhibits many different biological activities in development, inflammation, hematopoiesis, and cancer [24]. OSM receptor (usually found in literature as OSMRβ) is recruited after the initial interaction of OSM with gp130, completing the formation of the active complex that triggers intracellular signaling [25]. Alternatively, in humans, OSM can also induce cell responses by binding to LIFR/gp130 [26] (Figure 1C).

IL-11 was initially characterized in bone marrow-derived stromal cells as a hematopoietic cytokine with thrombopoietic activity [27]. In recent years, numerous studies have shown that IL-11 has a relevant role in breast cancer bone metastasis development [28]. IL-11 binds to IL-11 receptor α (IL-11Rα) and the subsequent interaction of this complex with gp130 triggers downstream signaling [29]. Cleavage of IL-11Rα produces a soluble form (sIL-11Rα), which is also able to bind IL-11 and activate trans-signaling specific responses [30] (Figure 1C).

For IL-6, LIF, OSM, and IL-11, cytokines binding to their membrane receptors leads to the activation of three main downstream pathways: JAK-STAT, Ras-Raf MEK/ERK, and PI3K/AKT signaling [31]. However, depending on the cell type, IL-6 family can trigger more than one cascade even simultaneously, affecting different functions. For example, these pleiotropic effects have been reported for LIF and OSM in trophoblast cells [32] and for another member of this family, the ciliary neurotrophic factor (CNTF), in human multipotent adipose-derived stem (hMADS) cells [33]. The multiplicity of their activities may explain the complex and sometimes contradictory effects that this family of proteins exerts on tumor and stromal cells and the difficulties to target them and/or their receptors for cancer treatment.

In 2020, breast cancer (BC) was the most commonly diagnosed cancer, with an estimated 2.3 million new cases worldwide, and the leading cause of cancer death for women [34]. Therefore, finding new therapeutic strategies for increasing survival rate of this illness is of utmost importance. Five intrinsic molecular BC subtypes have been described: Luminal A, Luminal B, HER2-enriched, Basal-like, and Claudin-low [35]. However, the last one is not analogous to the other four subtypes, but is a complex phenotype that can be found within the other categories [36]. Most Luminal A and B tumors express estrogen receptor (ER+) and often show good response to endocrine therapy, particularly the Luminal A subtype, when treated early. Most tumors in the HER2-Neu subtype are HER2+ and do not express high levels of neither ER nor progesterone receptors (ER− PR−). HER2-Neu breast cancers tend to grow fast and be invasive; however, specific anti-HER2 therapeutics, as trastuzumab, have improved survival rates of patients with this tumor subtype [35]. Finally, the Basal-like are triple-negative (ER− PR− HER2−) breast cancers (TNBC) that commonly display a very aggressive behavior. This subtype has less specific therapeutic options than the others and the worst prognosis among all BC categories [37].

Metastasis is the spread of neoplastic cells beyond the tissue of origin and, for breast cancer patients, is the main cause of mortality. The most common secondary sites for mammary tumor development are bone, lung, liver, and brain, and the capacity to invade these organs is not only due to cancer cell intrinsic features, but also to signals provided by the new microenvironment [38]. Epithelial to mesenchymal transition (EMT) of tumor cells facilitates their migration and invasiveness, and stromal cells, such as cancer associated fibroblasts (CAFs) and cancer associated adipocytes (CAAs) significantly contribute to the malignant phenotype in the primary and secondary sites by releasing specific signals [39], which include the IL-6 family of cytokines [40].

## 2. IL-6 in Breast Cancer

IL-6-induced signaling in breast tumors mainly triggers STAT3 activation. A positive correlation between phosphorylated STAT3 (pSTAT3) and IL-6 expression in primary breast tumors was first reported by Berishaj and collaborators, who also demonstrated that in vitro blockade of gp130 or IL-6 sequestration led to a decrease of pSTAT3 levels [41]. This report also demonstrated the capability of IL-6 containing conditioned media to stimulate STAT3 phosphorylation. Therefore, taking into account the dramatic role that this transcription factor plays in breast cancer progression [42], it was indubitable the significant participation of IL-6 in the development of this illness.

Involvement of IL-6 in breast cancer has been extensively reported over the years. Several groups from the 1980s to 2000s reported higher levels of this cytokine when sera from breast cancer patients and healthy women were compared [43]. Circulating IL-6 levels could be considered an independent prognostic marker given that higher IL-6 levels correlated with the stage of the disease [44]. Furthermore, in patients with untreated metastatic breast cancer, higher circulating IL-6 levels detected at the moment of diagnosis correlated with worse survival rates [45]. Conversely, a recent study shows that high levels of IL-6 and IL-10 detected in early stages of invasive breast cancer could be linked with good prognosis [46].

Several reports indicate that breast cancer cell lines produce and secrete IL6-family cytokines. Nevertheless, ER+ secrete lower IL-6 levels than ER− cells [47]. The first report on this observation goes up to 1996, when detectable levels of IL6 were found in the conditioned media of ER− but not ER+ human breast cancer cell lines [11]. It has been determined that ER inhibits IL-6 expression by disrupting NF-κB transactivation [48]. Furthermore, Chiu et al. had also shown that ER+ breast cancer cells express and secrete the soluble IL-6R, while ER− cells mainly express the receptor transmembrane isoform [11].

The role of IL-6 as a promoter of malignancy in breast cancer has been well established in different models and conditions. In 1989, a report was published demonstrating for the first time that IL-6 addition to cell culture medium enhanced motility as well as transition from cuboidal to fibroblastoid-like morphology of ER+ breast cancer cells. Importantly, these effects reverted upon cytokine removal [49]. A few years later another group shed some light on the involved mechanism demonstrating that incubation with IL-6 decreased E-cadherin expression in these cell lines [43]. Later studies confirmed that IL-6 increases migration and invasion capability of ER+ cells [50,51]. Therefore, it can be argued that even if ER+ breast cancer cells express low levels of IL-6, these cells can be highly susceptible to the presence of this cytokine in the microenvironment.

The association of IL-6 expression and breast cancer bad prognosis would not only be due to its relevance in tumor cell motility and epithelial-to-mesenchymal transition (EMT), but also to its essential role in cancer stem cell (CSC) self-renewal. It has been reported that IL-6 is upregulated in tumorspheres generated from patients’ aggressive ductal breast carcinomas and from ER+ cell lines. Addition of this cytokine to those organoids promoted hypoxia-resistance, self-renewal, and invasiveness [51]. Stem phenotype induction upon ectopic IL-6 addition has been also demonstrated in TNBC cell lines and in primary cultures from human breast samples [52].

The role of the microenvironment on tumor initiation and progression has been increasingly studied in the last years. IL-6 is not only expressed and secreted by breast cancer cells, but also by diverse cell types that are part of the tumor microenvironment, such as myeloid-derived suppressor cells (MDSCs) [53], fibroblasts [38,47], lymphatic endothelial cells [54], and adipocytes [55] (Figure 1A). Evidence of IL-6 release from a wide range of cell types and its association with breast cancer progression has been confirmed over the years and it has been described in different review articles [31,56]. Particularly, the contribution of adipose tissue has called the attention of various authors, since IL-6 serum levels positively correlated with body mass in obese breast cancer patients [57]. This finding is especially relevant considering that survival rates of obese women with breast cancer are lower than those of non-obese breast cancer patients with similar tumor grades [58]. Besides, more advanced and higher-grade tumors exhibited increased IL-6 levels in the peritumoral adipose tissue [55]. In vitro studies support these clinically observed correlations, and it has now been established that adipocyte secreted IL-6 promotes migration of ER+ and ER− tumor cells [59].

Endocrine therapy (ET)—based on the use of tamoxifen, fulvestrant, and aromatase inhibitors—is the standard treatment for patients with ER+ breast tumors. However, in spite of its initial benefit, later resistance is very common and represents a hard clinical challenge [60]. Over the years, IL-6 has been increasingly linked to the acquisition of ET resistance in breast cancer patients [61]. Interestingly, ET resistant self-renewing CSCs from luminal tumors were characterized by the display of CD133hi/ERlo/IL6hi markers. These cells were IL-6/Notch dependent, and inhibition of these pathways induced re-expression of the ER and recovery of ET sensitivity [51].

IL-6 has been also implicated in resistance to trastuzumab treatment for women with HER-2/Neu breast cancer subtype. In these tumors it has been reported the induction of an IL-6 inflammatory feedback loop that leads to the expansion of CSCs, which in turn secrete high levels of this cytokine. Importantly, addition of tocilizumab, an anti-IL-6R antibody, is sufficient to revert this effect in vivo leading to tumor and metastasis inhibition [62]. Based on these data, a treatment that combines trastuzumab with tocilizumab is currently in a Phase I clinical trial for patients with metastatic trastuzumab-resistant HER2+ breast cancer (NCT03135171).

TNBC is defined by the lack of ER and PR, as well as low HER2 expression. Consequently, treatments for this BC subtype are limited to radiation therapy or chemotherapy [63]. In TNBC cell lines, inhibition of IL-6 and IL-8 expression dramatically reduced colony formation and cell survival in vitro and prevented tumor engraftment and growth in vivo [64]. Growth of TNBC xenografts has been inhibited by tocilizumab [54]. Interestingly, patients with TNBC showed increased presence of tumor associated macrophages and higher expression levels of IL-6 after surgery [65].

## 3. LIF in Breast Cancer

It has been shown that LIF and OSM display higher expression levels in advanced breast cancer compared with benign or in situ lesions. Therefore, it has been proposed that higher levels of these cytokines, secreted either by mammary parenchyma or stromal cells, promote tumor growth [66]. Similarly to what was indicated above for IL-6 [47], ER− PR− cells, as MDA-MB231, have shown higher LIF expression levels than luminal tumor cell lines, as MCF7, MDA-MB468, or T47D [67]. It has been demonstrated that LIF promotes breast cancer cell proliferation in culture and in vivo [68], as well as EMT in MCF-7 and T47D tumor cells, and knockdown of endogenous LIF have reversed EMT in MDA-MB231 cells [67].

It has been reported that LIF oncogenic activity is mediated by STAT3 phosphorylation and translocation to the nuclei, and our group has reported that activation of this transcription factor in mammary tumor cells is dependent on paracrine/autocrine LIF secretion [69]. Interestingly, the rise of LIF in tumor microenvironment would be due to the presence of transforming growth factor β (TGF-β) that induces LIF production in both stroma and parenchyma cells. In turn, this would trigger the JAK/STAT signaling pathway that is able to activate tumor cell invasiveness [70]. In addition, it has been proposed that LIF activation of the JAK/STAT3 pathway also leads to tumorigenic miR-21 expression [67]. However, other cascades have been reported to mediate LIF activity in tumorigenesis and metastasis development, as the AKT/mTOR signaling pathway [68].

In spite of the data indicating the pro-oncogenic role of LIF, a whole genome RNAi screening identified LIFR as a mammary tumor suppressor [71]. Data analysis from a cohort of non-metastatic breast tumors has revealed that high LIFR expression levels correlate with increased metastasis-free, recurrence-free, and overall survival rates. Furthermore, LIFR expression restoration in malignant tumor cells triggered the Hippo signaling that led to functional inactivation of oncogenic YAP [72]. Besides, LIFR has been shown to be downregulated in patients with poorer prognosis among a cohort of breast cancer patients with bone metastasis [73]. Supporting these findings, it has been reported that breast cancer cells with low metastatic potential, such as MCF-7, express functional LIFR, but breast cancer cells that aggressively colonize the lung or bone, like MDA-MB231, lack a functional LIFR [31]. Finally, LIFR signaling blockade by hypoxia may allow breast cancer cells to exit dormancy in the bone marrow to proliferate and give rise to metastasis [73].

Specific inhibition of LIF/LIFR interactions has been proposed as a promising target for breast cancer therapy [74,75,76]. For treatment of solid tumors, such as breast cancer, the efficacy of histone deacetylase inhibitors (HDACs) has been suggested to be limited by LIFR expression induction and the consequent pro-oncogenic activation of the JAK/STAT3 signaling pathway. Therefore, combining HDAC with JAK1 or BRD4 (which is recruited at LIFR gene promoter in cells under HDAC treatment) blockers has been proposed for breast cancer treatment [74]. In addition, it has been shown that rapamycin, an inhibitor of the mTOR pathway, suppresses the promoting effect of LIF on tumorigenesis and metastasis in breast cancer cells [64] and that combined immunization against LIF and LIFR inhibits tumor formation from mammary CSCs in BALB/c mice [77]. Additionally, a humanized anti-LIF antibody, MSC-1, has recently been developed and is being tested as a cancer therapy in a phase I clinical trial for advanced metastatic solid tumors (ClinicalTrials.gov: NCT03490669) [75].

## 4. OSM in Breast Cancer

OSM has been reported to induce migration, invasiveness [78,79], and loss of hormone receptor expression in human breast cancer cells [80]. Furthermore, high expression of OSM and its receptor (OSMR) was associated with poor prognosis for breast cancer patients [81,82]. This cytokine has also promoted CSC plasticity through STAT3 activation [83]. OSM treatment have induced EMT and stemness associated features in luminal breast cancer cell lines [82], as well as acquisition of stem capabilities in TNBC cells through repression of both autocrine and paracrine IFN-β signaling [84]. In addition, it has been reported that OSM together with IL-1β induces IL-6 in breast cancer cells in culture, and expression of these three factors correlated with low breast cancer patient survival. Therefore, breast cancer treatment regimens that simultaneously suppress multiple cytokines with overlapping functions have been proposed to increase patient survival [81].

In a mouse model, it has been determined that OSM produced by mammary tumor cells promoted tumor osteolytic bone metastasis through amphiregulin (AREG) induction, which led to osteoclast activation [85]. It has been reported that in mice bearing human breast tumor xenografts, peritumoral injection of OSM increased the number of metastases to the lung, decreased mouse survival, and augmented the quantity of circulating tumor cells (CTC). All these effects were reduced in OSM-KO mice bearing the same tumors [86]. Therefore, these authors proposed that early treatment of breast cancer patients with OSM inhibitors may prevent metastatic progression.

Based on the evidence summarized above, the use of OSM monoclonal antibodies that have been already tested in humans [87] is a promising possibility for breast cancer patients.

## 5. IL-11 in Breast Cancer

Expression analysis of IL-11 and its receptor in human breast cancers revealed that their high levels are clinically associated with lower survival [88]. Mechanistically, it has been proposed that this link would be due, at least partially, to the decrease of the tumor suppressor microRNA-30c (miR-30c), which prevents tumor chemotherapy resistance by directly reducing twinfilin 1 (TWF1) levels and its secondary target, secreted IL-11 [89]. Similarly, hsa-miR-206, which inhibits self-renewal and invasiveness of breast cancer cells, also targets TWF1, which enhances MKL1 and SRF that in turn, promoting IL-11 expression. As it has been demonstrated that the MKL1-SRF/IL-11 signaling is essential for miR-206 function in regulating breast cancer stem cells and tumorigenesis, analyzing this pathway may lead to innovative drug development to control cancer growth and prevent metastasis [90].

High IL-11 expression has been particularly observed in breast cancer patients with bone metastasis [90,91,92] and the detection of IL-11 in primary breast cancer has been proposed as a predictive factor for tumor growth in that secondary site [93]. Besides, it has been determined that parathyroid hormone-related peptide (PTHrP) secreted by breast cancer cells invading the bone induces IL-11 by host osteoblasts that sequentially exerts osteoclastogenic activity through PGE2 increase [94]. However, IL-11 expression by breast cancer cells would also be relevant in determining bone metastatic ability as it has been reported that the small group of cells in a human breast cancer line that possessed that capacity expressed a specific gene profile, which included over-expression of this cytokine. Interestingly, none of the genes in the bone metastasis signature were part of those previously described in the poor-prognosis breast cancer profile [95].

Aberrantly activated NRF2 in cancer cells may drive malignant progression and it has been reported that IL-11 is induced downstream of NRF2. In breast cancer patients, a positive correlation between IL-11 and NRF2 has been detected, although this association has not been detected in cultured breast cancer cells. These results imply that a signal originating from the microenvironment would cooperate with NRF2 to activate IL-11, and it has been demonstrated that transformed fibroblasts may provide a critical contribution of IL-11 to NRF2-dependent tumorigenesis. Therefore, these studies suggest that inhibition of IL-11 signaling would be an effective strategy for inhibiting progression of NRF2-addicted breast cancers [96].

## 6. IL-6 Cytokine Family in Post-Partum Breast Cancer

Women diagnosed with breast cancer within 5 to 10 years of childbirth have significantly increased risk for metastatic recurrence [97,98]. It has been proposed that the higher probability of developing a metastatic disease during that period would be caused by mammary gland involution. During this process, which starts when milk production ends, either after birth in the absence of nursing or after weaning, dramatic tissue remodeling occurs in the mammary gland of all female mammals [99,100].

In female mice, expression of IL-6, LIF and OSM is low during lactation, but increases during involution together with the activation of Stat3 in mammary tissue. It has been shown that LIF is the cytokine responsible for activation of this transcription factor [101,102], while IL-6 induced ERK1/2 MAPK phosphorylation, which also plays a significant role during mammary regression [103]. Furthermore, OSM and OSMR are upregulated in response to STAT3 activation and the signaling triggered by this cytokine promoted the expression of metalloproteinases MMP3, MMP12, and MMP14, that are relevant for remodeling normal mammary tissue, but would also facilitate mammary tumor invasiveness [104].

Transgenic mice with mammary specific expression of the protease pappalysin-1 (PAPP-A), which is extensively overexpressed in breast cancers [105], have shown that extended lactation is protective against the oncogenic effect of PAPP-A, while abrupt halt of nursing increased its tumorigenic impact [106]. Interestingly, during mammary involution, PAPP-A expression is induced by IL-6 among other cytokines, and sudden interruption of lactation have triggered an increase in inflammation markers [107]. Therefore, possibly by ameliorating inflammation in the post-partum mammary microenvironment, extended lactation may be protective against breast cancer via suppression of PAPP-A [108].

## 7. IL-6 Cytokine Family in Mastitis

Mastitis, an inflammation of the mammary gland, is a worrying condition for nursing women, which may or may not be accompanied by infection. It is characterized by breast pain, swelling as well as redness, and can be followed by serious complications such as abscess and septicemia. Studies in different western countries revealed a relevant impact of this pathology with incidences of about 10–20% during the first 3 months post-delivery [109]. This pathology has also a great impact on cow milk production, since it causes premature weaning leading to reduced milk yield and quality. In addition, mastitis increases the cost of cattle management together with danger of antibiotic residues in commercialized milk [110]. This is due to the fact that in dairy cows, mastitis is usually caused by different bacteria including Gram-positive, e.g., *Staphylococcus aureus* (*S. aureus*) and Gram-negative, such as *Escherichia coli* (*E. coli*) [111]. The pathogenesis caused by *E. coli* and other Gram-negative bacteria is often characterized by an acute inflammation, which, however, may eventually lead to pathogen clearance [112]. On the other hand, Staphylococci are the bacteria most commonly isolated from cases of subclinical mastitis [113]. Infection with these Gram-positive pathogens often causes mild signs of mastitis, but ineffective pathogen clearance frequently leads to chronic infection [114]. Importantly, infections by *S. aureus* are also a serious problem for women, because antibiotic resistance may cause eventual severity and difficulties to cure the illness [115].

Analysis of the transcriptome of primary bovine mammary epithelial cells after challenging them with heat-inactivated preparations of *E. coli* or *S. aureus* showed that the first rapidly and strongly induced expression of cytokines and chemokines while *S. aureus* elicited a retarded response. The genes that were most strongly upregulated by *E. coli* were clustered into a regulatory network with tumor necrosis factor alpha (TNF-α) and interleukin-1 (IL-1) in a central position. In contrast, the *S. aureus* response induced a functional network dominated by IL-6, although this cytokine would be also relevant for *E. coli* late response. Therefore, detection of IL-6 in milk has been proposed as a predictor marker for subclinical mastitis [116].

Interestingly, not only IL-6—but also its receptor, IL-6R—has been reported to be upregulated in the mammary glands of mastitic cows. This enhanced expression has been attributed to increased DNA methylation level during inflammation. Specifically, it has been proposed that the DNA methylation level in exon 2 of IL-6R could also be a potential biomarker for monitoring bovine mastitis [117]. Furthermore, Zhu et al., demonstrated an increase in the IL-6 mRNA in infected mammary lobes, but not in the mammary lobes that did not develop mastitis [118]. Similarly, comparing protein content in breast milk from human nipple single pores revealed that milk from mastitic lobes contained higher concentration of IL-6 than milk from healthy glands [119].

Plasma cell mastitis (PCM), also known as mammary ductal ectasia, is a special form of mastitis that typically occurs in young and middle-aged women at nonpregnant or non-nursing stages. IL-6/STAT3 signaling is activated in PCM and may play an important role in the pathogenesis of this illness [120]. Associated with this discovery, it has been proposed that sinomenine hydrochloride may achieve a therapeutic effect on PCM based on its anti-inflammatory and immunoregulatory properties, which are exerted by IL-6/JAK2/STAT3 pathway downregulation [121].

## 8. Concluding Remarks

The data reviewed here clearly show that IL-6 cytokine family plays an important role in breast cancer progression (Table 1). Several studies have demonstrated the involvement of these factors in the cross talk between tumor cells and their microenvironment in the primary as well as in secondary sites, which has a dramatic impact on treatment resistance and cancer relapse. Targeting the IL-6 cytokine family/JAK/STAT-3 pathway may be a fruitful approach for early adjuvant breast cancer therapy and/or for preventing aggressive development of metastasis in secondary sites. This signaling cascade can be targeted in multiple ways, such as the use of specific monoclonal antibodies against the cytokines or their receptors, or by using synthetic/semi-synthetic compounds as specific inhibitors of downstream signaling molecules. Particularly in TNBC, to which therapeutic approaches are scarce compared with the other subtypes, high levels of IL-6 have been reported and pre-clinical analysis of IL-6 inhibition have been published. Therefore, with the approval of immunotherapy for this subtype [122], it is worth considering the mechanistic link found between IL-6 and PDL1 [123] to be applied in breast cancer treatment. For the ER+ and HER2-Neu breast cancer subtypes, IL-6 has been increasingly linked to acquisition of resistance and escape from specific therapies applied in these subtypes. Therefore, strategies combining endocrine therapy or HER2-Neu blockade with IL-6 pathway inhibition would be plausible therapeutic approaches to significantly improve relapse-free survival of patients diagnosed with these breast cancer subtypes.

## Figures and Tables

**Table 1:** Comparative summary. Most relevant studies, cited in this review, about IL-6 cytokine family role in breast cancer development and treatment.

Topic	Cytokines [Refs]
Stat-3 phosphorylation induction	IL-6 [41]; LIF [67,69,70]; OSM [83]
High expression and/or circulating levels are associated with cancer stage or prognosis	IL-6 [44,45]; LIF [66]; OSM [66,81,82]; IL-11 [88]
ER+ breast cancer cells express and/or secrete lower cytokine levels than ER− cells	IL-6 [11,47,48]; LIF [67]
CSC maintenance, EMT, cell migration and/or invasion induction	IL-6 [43,49,50,51,52]; LIF [67]; OSM [78,79,82,83,84]
Expression by diverse cell types in tumor microenvironment	IL-6 [31,38,47,53,54,55,56,57,59]; IL-11 [94,96]
Association with Her2 or endocrine therapy resistance	IL-6 [61,62]
Induction of TNBC progression	IL-6 [54,64,65]
Use in targeted therapy	IL-6 [54,62]; LIF [74,75,76,77]; OSM [87]
Receptor as tumor suppressor	LIF [71,72,73]
Tumor miRNA modulation	LIF [67]; IL-11 [89,90]
Involvement in metastatic progression	OSM [85,86]; IL-11 [91,92,93,94,95]

## Data Availability

Not applicable.

## Abbreviations

BC	Breast Cancer
CAAs	Cancer Associated Adipocytes,
CAFs	Cancer Associated Fibroblasts
CSC	Cancer Stem Cells
CTCs	Circulating Tumor Cells
EMT	Epithelial-to-Mesenchymal Transition
ER	Estrogen Receptor
ET	Endocrine Therapy
gp130	Glycoprotein130
HER2	Human Epidermal Growth Factor Receptor 2
IL-11	Interleukin-11
IL-11R	Interleukin-11 Receptor
IL-6	Interleukin-6
IL-6R	Interleukin-6 Receptor
LECs	Lymphatic Endothelial Cells
LIF	Leukemia Inhibitory Factor
LIFR	LIF Receptor
MDSCs	Myeloid Derived Stem Cells
miR	Micro-RNA
OSM	Oncostatin M
OSMR	Oncostatin M Receptor
PR	Progesterone Receptor
pSTAT·	Phosphorylated STAT3
TNBC	Triple Negative Breast Cancer

## References

[1] Bravo J.K., Heath J.K. (2000). Receptor recognition by gp130 cytokines. EMBO J..

[2] Boulanger M.J., Garcia K.C. (2004). Shared cytokine signaling receptors: Structural insights from the gp130 system. Adv. Protein Chem..

[3] Kang S., Tanaka T., Narazaki M., Kishimoto T. (2019). Targeting Interleukin-6 Signaling in Clinic. Immunity.

[4] Taga T., Hibi M., Hirata Y., Yamasaki K., Yasukawa K., Matsuda T., Hirano T., Kishimoto T. (1989). Interleukin-6 triggers the association of its receptor with a possible signal transducer, gp130. Cell.

[5] Hirano T., T Yasukawa K., Harada H., Taga T., Watanabe Y., Matsuda T., Kashiwamura S., Nakajima K., Koyama K., Iwamatsu A. (1986). Complementary DNA for a novel human interleukin (BSF-2) that induces B lymphocytes to produce immunoglobulin. Nature.

[6] Potter M., Boyce C.R. (1962). Induction of plasma-cell neoplasms in strain BALB/c mice with mineral oil and mineral oil adjuvants. Nature.

[7] Gauldie J., Richards C., Harnish D., Lansdorp P., Baumann H. (1987). Interferon beta 2/B-cell stimulatory factor type 2 shares identity with monocyte-derived hepatocyte-stimulating factor and regulates the major acute phase protein response in liver cells. Proc. Natl. Acad. Sci. USA.

[8] Tanaka T., Narazaki M., Kishimoto T. (2014). IL-6 in inflammation, immunity, and disease. Cold Spring Harb. Perspect. Biol..

[9] Boulanger M.J., Chow D.C., Brevnova E.E., Garcia K.C. (2003). Hexameric structure and assembly of the interieukin-6/IL-6 α-recepor/gp130 complex. Science.

[10] Saito M., Yoshida K., Hibi M., Taga T., Kishimoto T. (1992). Molecular cloning of a murine IL-6 receptor-associated signal transducer, gp130, and its regulated expression in vivo. J. Immunol..

[11] Chiu J.J., Sgagias M.K., Cowan K.H. (1996). Interleukin 6 acts as a paracrine growth factor in human mammary carcinoma cell lines. Clin. Cancer Res..

[12] Jostock T., Müllberg J., Ozbek S., Atreya R., Blinn G., Voltz N., Fischer M., Neurath M.F., Rose-John S. (2001). Soluble gp130 is the natural inhibitor of soluble interleukin-6 receptor transsignaling responses. Eur. J. Biochem..

[13] Rose-John S., Heinrich P.C. (1994). Soluble receptors for cytokines and growth factors: Generation and biological function. Biochem. J..

[14] Rose-John S. (2012). Il-6 trans-signaling via the soluble IL-6 receptor: Importance for the proinflammatory activities of IL-6. Int. J. Biol. Sci..

[15] Gearing D.P., Gough N.M., King J.A., Hilton D.J., Nicola N.A., Simpson R.J., Nice E.C., Kelso A., Metcalf D. (1987). Molecular cloning and expression of cDNA encoding a murine myeloid leukaemia inhibitory factor (LIF). EMBO J..

[16] Metcalfe S.M. (2011). LIF in the regulation of T-cell fate and as a potential therapeutic. Genes Immun..

[17] Hirai H., Karian P., Kikyo N. (2011). Regulation of embryonic stem cell self-renewal and pluripotency by leukaemia inhibitory factor. Biochem. J..

[18] Gearing D.P., Thut C.J., VandeBos T., Gimpel S.D., Delaney P.B., King J., Price V., Cosman D., Beckmann M.P. (1991). Leukemia inhibitory factor receptor is structurally related to the IL-6 signal transducer, gp130. EMBO J..

[19] Chambers I., Cozens A., Broadbent J., Robertson M., Lee M., Li M., Smith A. (1997). Structure of the mouse leukaemia inhibitory factor receptor gene: Regulated expression of mRNA encoding a soluble receptor isoform from an alternative 5′ untranslated region. Biochem. J..

[20] Tomida M. (2000). Structural and functional studies on the leukemia inhibitory factor receptor (LIF-R): Gene and soluble form of LIF-R, and cytoplasmic domain of LIF-R required for differentiation and growth arrest of myeloid leukemic cells. Leuk. Lymphoma.

[21] Metz S., Naeth G., Heinrich P.C., Müller-Newen G. (2008). Novel inhibitors for murine and human leukemia inhibitory factor based on fused soluble receptors. J. Biol. Chem..

[22] Nicola N.A., Babon J.J. (2015). Leukemia inhibitory factor (LIF). Cytokine Growth Factor Rev..

[23] Zarling J.M., Shoyab M., Marquardt H., Hanson M.B., Lioubin M.N., Todaro G.J. (1986). Oncostatin M: A growth regulator produced by differentiated histiocytic lymphoma cells. Proc. Natl. Acad. Sci. USA.

[24] Tanaka M., Miyahima A. (2003). Oncostatin M, a multifunctional cytokine. Rev. Physiol. Biochem. Pharmacol..

[25] Deller M.C., Hudson K.R., Ikemizu S., Bravo J., Jones E.Y., Heath J.K. (2000). Crystal structure and functional dissection of the cytostatic cytokine oncostatin M. Structure.

[26] Gearing D.P., Comeau M.R., Friend D.J., Gimpel S.D., Thut C. J., McGourty J., Brasher K. K., King J. A., Gillis S., Mosley B. (1992). The IL-6 signal transducer, gp130: An oncostatin M receptor and affinity converter for the LIF receptor. Science.

[27] Paul S.R., Bennett F., Calvetti J.A., Kelleher K., Wood C.R., O’Hara R.M., Leary A.C., Sibley B., Clark S.C., Williams D.A. (1990). Molecular cloning of a cDNA encoding interleukin 11, a stromal cell-derived lymphopoietic and hematopoietic cytokine. Proc. Natl. Acad. Sci. USA.

[28] Maroni P., Bendinelli P., Ferraretto A., Lombardi G. (2021). Interleukin 11 (IL-11): Role(s) in Breast Cancer Bone Metastases. Biomedicines.

[29] Permyakov E.A., Uversky V.N., Permyakov S.E. (2016). Interleukin-11: A Multifunctional Cytokine with Intrinsically Disordered Regions. Cell Biochem. Biophys..

[30] Lokau J., Nitz R., Agthe M., Monhasery N., Aparicio-Siegmund S., Schumacher N., Wolf J., Möller-Hackbarth K., Waetzig G.H., Grötzinger J. (2016). Proteolytic Cleavage Governs Interleukin-11 Trans-signaling. Cell Rep..

[31] Omokehinde T., Johnson R.W. (2020). Gp130 cytokines in breast cancer and bone. Cancers.

[32] Chaiwangyen W., Ospina-Prieto S., Morales-Prieto D.M., Pereira de Sousa F.L., Pastuschek J., Fitzgerald J.S., Schleussner E., Markert U.R. (2016). Oncostatin M and leukaemia inhibitory factor trigger signal transducer and activator of transcription 3 and extracellular signal-regulated kinase 1/2 pathways but result in heterogeneous cellular responses in trophoblast cells. Reprod. Fertil. Dev..

[33] Perugini J., Di Mercurio E., Tosseta G., Severi I., Monaco F., Reguzzoni M., Tomasetti M., Dani C., Cinti S., Giordano A. (2019). Biological Effects of Ciliary Neurotrophic Factor on hMADS Adipocytes. Front. Endocrinol..

[34] Sung H., Ferlay J., Siegel R.L., Laversanne M., Soerjomataram I., Jemal A., Bray F. (2021). Global Cancer Statistics 2020: GLOBOCAN Estimates of Incidence and Mortality Worldwide for 36 Cancers in 185 Countries. CA Cancer J. Clin..

[35] Prat A., Pineda E., Adamo B., Galván P., Fernández A., Gaba L., Díez M., Viladot M., Arance A., Muñoz M. (2015). Clinical implications of the intrinsic molecular subtypes of breast cancer. Breast.

[36] Fougner C., Bergholtz H., Norum J.H., Sørlie T. (2020). Re-definition of claudin-low as a breast cancer phenotype. Nat. Commun..

[37] Nishimura R., Arima N. (2008). Is triple negative a prognostic factor in breast cancer?. Breast Cancer.

[38] Studebaker A.W., Storci G., Werbeck J.L., Sansone P., Sasser A.K., Tavolari S., Huang T., Chan M.W., Marini F.C., Rosol T.J. (2008). Fibroblasts Isolated from Common Sites of Breast Cancer Metastasis Enhance Cancer Cell Growth Rates and Invasiveness in an Interleukin-6–Dependent Manner. Cancer Res..

[39] Hanahan D., Weinberg R.A. (2011). Hallmarks of cancer: The next generation. Cell.

[40] Jones S.A., Jenkins B.J. (2018). Recent insights into targeting the IL-6 cytokine family in inflammatory diseases and cancer. Nat. Rev. Immunol..

[41] Berishaj M., Gao S.P., Ahmed S., Leslie K., Al-Ahmadie H., Gerald W.L., Bornmann W., Bromberg J.F. (2007). Stat3 is tyrosine-phosphorylated through the interleukin-6/glycoprotein 130/Janus kinase pathway in breast cancer. Breast Cancer Res..

[42] Hughes K., Watson C.J. (2018). The multifaceted role of STAT3 in mammary gland involution and breast cancer. Int. J. Mol. Sci..

[43] Ásgeirsson K.S., Ólafsdóttir K., Jónasson J.G., Ógmundsdóttir H.M. (1998). The effects of IL-6 on cell adhesion and E-cadherin expression in breast cancer. Cytokine.

[44] Kozłowski L., Zakrzewska I., Tokajuk P., Wojtukiewicz M.Z. (2003). Concentration of interleukin-6 (IL-6), interleukin-8 (IL-8) and interleukin-10 (IL-10) in blood serum of breast cancer patients. Rocz. Akad. Med. Białymstoku.

[45] Salgado R., Junius S., Benoy I., Van Dam P., Vermeulen P., Van Marck E., Huget P., Dirix L.Y. (2003). Circulating interleukin-6 predicts survival in patients with metastatic breast cancer. Int. J. Cancer.

[46] Ahmad N., Ammar A., Storr S.J., Green A.R., Rakha E., Ellis I.O., Martin S.G. (2018). IL-6 and IL-10 are associated with good prognosis in early stage invasive breast cancer patients. Cancer Immunol. Immunother..

[47] Sasser A.K., Sullivan N.J., Studebaker A.W., Hendey L.F., Axel A.E., Hall B.M. (2007). Interleukin-6 is a potent growth factor for ER-α-positive human breast cancer. FASEB J..

[48] Liu H., Liu K., Bodenner D.L. (2005). Estrogen receptor inhibits interleukin-6 gene expression by disruption of nuclear factor kappaB transactivation. Cytokine.

[49] Tamm I., Cardinale I., Krueger J., Murphy J.S., May L.T., Sehgal P.B. (1989). Interleukin 6 decreases cell-cell association and increases motility of ductal breast carcinoma cells. J. Exp. Med..

[50] Badache A., Hynes N.E. (2001). Interleukin 6 Inhibits Proliferation and, in Cooperation with an Epidermal Growth Factor Receptor Autocrine Loop, Increases Migration of T47D Breast Cancer Cells 1. Cancer Res..

[51] Sansone P., Storci G., Tavolari S., Guarnieri T., Giovannini C., Taffurelli M., Ceccarelli C., Santini D., Paterini P., Marcu K.B. (2007). IL-6 triggers malignant features in mammospheres from human ductal breast carcinoma and normal mammary gland. Am. Soc. Clin. Investig..

[52] Iliopoulos D., Hirsch H.A., Wang G., Struhl K. (2011). Inducible formation of breast cancer stem cells and their dynamic equilibrium with non-stem cancer cells via IL6 secretion. Proc. Natl. Acad. Sci. USA.

[53] Oh K., Lee O.Y., Shon S.Y., Nam O., Ryu P.M., Seo M.W., Lee D.S. (2013). A mutual activation loop between breast cancer cells and myeloid-derived suppressor cells facilitates spontaneous metastasis through IL-6 trans-signaling in a murine model. Breast Cancer Res..

[54] Jin K., Pandey N.B., Popel A.S. (2018). Simultaneous blockade of IL-6 and CCL5 signaling for synergistic inhibition of triple-negative breast cancer growth and metastasis. Breast Cancer Res..

[55] Dirat B., Bochet L., Dabek M., Daviaud D., Dauvillier S., Majed B., Wang Y.Y., Meulle A., Salles B., Le Gonidec S. (2011). Cancer-associated adipocytes exhibit an activated phenotype and contribute to breast cancer invasion. Cancer Res..

[56] Gyamfi J., Eom M., Koo J.S., Choi J. (2018). Multifaceted Roles of Interleukin-6 in Adipocyte–Breast Cancer Cell Interaction. Transl. Oncol..

[57] Hoene M., Weigert C. (2008). The role of interleukin-6 in insulin resistance, body fat distribution and energy balance. Obes. Rev..

[58] Knüpfer H., Preiß R. (2007). Significance of interleukin-6 (IL-6) in breast cancer (review). Breast Cancer Res. Treat..

[59] Walter M., Liang S., Ghosh S., Hornsby P.J., Li R. (2009). Interleukin 6 secreted from adipose stromal cells promotes migration and invasion of breast cancer cells. Oncogene.

[60] Matutino A., Joy A.A., Brezden-Masley C., Chia S., Verma S. (2018). Hormone receptor-positive, HER2-negative metastatic breast cancer: Redrawing the lines. Curr. Oncol..

[61] Masjedi A., Hashemi V., Hojjat-Farsangi M., Ghalamfarsa G., Azizi G., Yousefi M., Jadidi-Niaragh F. (2018). The significant role of interleukin-6 and its signaling pathway in the immunopathogenesis and treatment of breast cancer. Biomed. Pharmacother..

[62] Korkaya H., Kim G.I., Davis A., Malik F., Henry N.L., Ithimakin S., Quraishi A.A., Tawakkol N., D’Angelo R., Paulson A.K. (2012). Activation of an IL6 Inflammatory Loop Mediates Trastuzumab Resistance in HER2+ Breast Cancer by Expanding the Cancer Stem Cell Population. Mol. Cell.

[63] Santoni M., Romagnoli E., Saladino T., Foghini L., Guarino S., Capponi M., Giannini M., Cognigni P.D., Ferrara G., Battelli N. (2018). Triple negative breast cancer: Key role of tumor-associated macrophages in regulating the activity of anti-PD-1/PD-L1 agents. Biochim. Biophys. Acta Rev. Cancer.

[64] Hartman Z.C., Poage G.M., den Hollander P., Tsimelzon A., Hill J., Panupinthu N., Zhang Y., Mazumdar A., Hilsenbeck S.G., Mills G.B. (2013). Growth of triple-negative breast cancer cells relies upon coordinate autocrine expression of the proinflammatory cytokines IL-6 and IL-8. Cancer Res..

[65] Liang S., Chen Z., Jiang G., Zhou Y., Liu Q., Su Q., Wei W., Du J., Wang H. (2017). Activation of GPER suppresses migration and angiogenesis of triple negative breast cancer via inhibition of NF-κB/IL-6 signals. Cancer Lett..

[66] García-Tuñón I., Ricote M., Ruiz A., Fraile B., Paniagua R., Royuela M. (2008). OSM, LIF, its receptors, and its relationship with the malignance in human breast carcinoma (in situ and in infiltrative). Cancer Investig..

[67] Yue X., Zhao Y., Zhang C., Li J., Liu Z., Liu J., Hu W. (2016). Leukemia inhibitory factor promotes EMT through STAT3-dependent miR-21 induction. Oncotarget.

[68] Li X., Yang Q., Yu H., Wu L., Zhao Y., Zhang C., Yue X., Liu Z., Wu H., Haffty B.G. (2014). LIF promotes tumorigenesis and metastasis of breast cancer through the AKT-mTOR pathway. Oncotarget.

[69] Quaglino A., Schere-Levy C., Romorini L., Meiss R.P., Kordon E.C. (2007). Mouse mammary tumors display Stat3 activation dependent on leukemia inhibitory factor signaling. Breast Cancer Res..

[70] Albrengues J., Bourget I., Pons C., Butet V., Hofman P., Tartare-Deckert S., Feral C.C., Meneguzzi G., Gaggioli C. (2014). LIF mediates proinvasive activation of stromal fibroblasts in cancer. Cell Rep..

[71] Iorns E., Ward T.M., Dean S., Jegg A., Thomas D., Murugaesu N., Sims D., Mitsopoulos C., Fenwick K., Kozarewa I. (2012). Whole genome in vivo RNAi screening identifies the leukemia inhibitory factor receptor as a novel breast tumor suppressor. Breast Cancer Res. Treat..

[72] Chen D., Sun Y., Wei Y., Zhang P., Rezaeian A.H., Teruya-Feldstein J., Gupta S., Liang H., Lin H.K., Hung M.C. (2012). LIFR is a breast cancer metastasis suppressor upstream of the Hippo-YAP pathway and a prognostic marker. Nat. Med..

[73] Johnson R.W., Finger E.C., Olcina M.M., Vilalta M., Aguilera T., Miao Y., Merkel A.R., Johnson J.R., Sterling J.A., Wu J.Y. (2016). Induction of LIFR confers a dormancy phenotype in breast cancer cells disseminated to the bone marrow. Nat. Cell Biol..

[74] Zeng H., Qu J., Jin N., Xu J., Lin C., Chen Y., Yang X., He X., Tang S., Lan X. (2016). Feedback Activation of Leukemia Inhibitory Factor Receptor Limits Response to Histone Deacetylase Inhibitors in Breast Cancer. Cancer Cell.

[75] Shi Y., Hunter S., Hunter T. (2019). Stem Cell Factor LIFted as a Promising Clinical Target for Cancer Therapy. Mol. Cancer Ther..

[76] Viswanadhapalli S., Luo Y., Sareddy G.R., Santhamma B., Zhou M., Li M., Ma S., Sonavane R., Pratap U.P., Altwegg K.A. (2019). EC359: A First-in-Class Small-Molecule Inhibitor for Targeting Oncogenic LIFR Signaling in Triple-Negative Breast Cancer. Mol. Cancer Ther..

[77] Ghanei Z., Mehri N., Jamshidizad A., Joupari M.D., Shamsara M. (2020). Immunization against leukemia inhibitory factor and its receptor suppresses tumor formation of breast cancer initiating cells in BALB/c mouse. Sci. Rep..

[78] Lapeire L., Hendrix A., Lambein K., Van Bockstal M., Braems G., Van Den Broecke R., Limame R., Mestdagh P., Vandesompele J., Vanhove C. (2014). Cancer-associated adipose tissue promotes breast cancer progression by paracrine oncostatin M and Jak/STAT3 signaling. Cancer Res..

[79] Holzer R.G., Ryan R.E., Tommack M., Schlekeway E., Jorcyk C.L. (2004). Oncostatin M stimulates the detachment of a reservoir of invasive mammary carcinoma cells: Role of cyclooxygenase-2. Clin. Exp. Metastasis.

[80] West N.R., Murphy L.C., Watson P.H. (2012). Oncostatin M suppresses oestrogen receptor-α expression and is associated with poor outcome in human breast cancer. Endocr.-Relat. Cancer.

[81] Tawara K., Scott H., Emathinger J., Wolf C., LaJoie D., Hedeen D., Bond L., Montgomery P., Jorcyk C. (2019). High expression of OSM and IL-6 are associated with decreased breast cancer survival: Synergistic induction of IL-6 secretion by OSM and IL-1β. Oncotarget.

[82] West N.R., Murray J.I., Watson P.H. (2014). Oncostatin-M promotes phenotypic changes associated with mesenchymal and stem cell-like differentiation in breast cancer. Oncogene.

[83] Junk D.J., Bryson B.L., Smigiel J.M., Parameswaran N., Bartel C.A., Jackson M.W. (2017). Oncostatin M promotes cancer cell plasticity through cooperative STAT3-SMAD3 signaling. Oncogene.

[84] Doherty M.R., Parvani J.G., Tamagno I., Junk D.J., Bryson B.L., Cheon H.J., Stark G.R., Jackson M.W. (2019). The opposing effects of interferon-beta and oncostatin-M as regulators of cancer stem cell plasticity in triple-negative breast cancer. Breast Cancer Res..

[85] Bolin C., Tawara K., Sutherland C., Redshaw J., Aranda P., Moselhy J., Anderson R., Jorcyk C.L. (2012). Oncostatin M promotes mammary tumor metastasis to bone and osteolytic bone degradation. Genes Cancer.

[86] Tawara K., Bolin C., Koncinsky J., Kadaba S., Covert H., Sutherland C., Bond L., Kronz J., Garbow J.R., Jorcyk C.L. (2018). OSM potentiates preintravasation events, increases CTC counts, and promotes breast cancer metastasis to the lung. Breast Cancer Res..

[87] Reid J., Zamuner S., Edwards K., Rumley S.A., Nevin K., Feeney M., Zecchin C., Fernando D., Wisniacki N. (2018). In vivo affinity and target engagement in skin and blood in a first-time-in-human study of an anti-oncostatin M monoclonal antibody. Br. J. Clin. Pharmacol..

[88] Hanavadi S., Martin T.A., Watkins G., Mansel R.E., Jiang W.G. (2006). Expression of Interleukin 11 and Its Receptor and Their Prognostic Value in Human Breast Cancer. Ann. Surg. Oncol..

[89] Bockhorn J., Dalton R., Nwachukwu C., Huang S., Prat A., Yee K., Chang Y.F., Huo D., Wen Y., Swanson K.E. (2013). MicroRNA-30c inhibits human breast tumour chemotherapy resistance by regulating TWF1 and IL-11. Nat. Commun..

[90] Samaeekia R., Adorno-Cruz V., Bockhorn J., Chang Y.F., Huang S., Prat A., Ha N., Kibria G., Huo D., Zheng H. (2017). miR-206 Inhibits Stemness and Metastasis of Breast Cancer by Targeting MKL1/IL11 Pathway. Clin. Cancer Res. Off. J. Am. Assoc. Cancer Res..

[91] Ren L., Wang X., Dong Z., Liu J., Zhang S. (2013). Bone metastasis from breast cancer involves elevated IL-11 expression and the gp130/STAT3 pathway. Med. Oncol..

[92] Cosphiadi I., Atmakusumah T.D., Siregar N.C., Muthalib A., Harahap A., Mansyur M. (2018). Bone Metastasis in Advanced Breast Cancer: Analysis of Gene Expression Microarray. Clin. Breast Cancer.

[93] Sotiriou C., Lacroix M., Lespagnard L., Larsimont D., Paesmans M., Body J.J. (2001). Interleukins-6 and -11 expression in primary breast cancer and subsequent development of bone metastases. Cancer Lett..

[94] Morgan H., Tumber A., Hill P.A. (2004). Breast cancer cells induce osteoclast formation by stimulating host IL-11 production and downregulating granulocyte/macrophage colony-stimulating factor. Int. J. Cancer.

[95] Kang Y., Siegel P.M., Shu W., Drobnjak M., Kakonen S.M., Cordón-Cardo C., Guise T.A., Massagué J. (2003). A multigenic program mediating breast cancer metastasis to bone. Cancer Cell.

[96] Kitamura H., Onodera Y., Murakami S., Suzuki T., Motohashi H. (2017). IL-11 contribution to tumorigenesis in an NRF2 addiction cancer model. Oncogene.

[97] Callihan E.B., Gao D., Jindal S., Lyons T.R., Manthey E., Edgerton S., Urquhart A., Schedin P., Borges V.F. (2013). Postpartum diagnosis demonstrates a high risk for metastasis and merits an expanded definition of pregnancy-associated breast cancer. Breast Cancer Res. Treat..

[98] Goddard E.T., Bassale S., Schedin T., Jindal S., Johnston J., Cabral E., Latour E., Lyons T.R., Mori M., Schedin P.J. (2019). Association Between Postpartum Breast Cancer Diagnosis and Metastasis and the Clinical Features Underlying Risk. JAMA Netw. Open.

[99] Jindal S., Gao D., Bell P., Albrektsen G., Edgerton S.M., Ambrosone C.B., Thor A.D., Borges V.F., Schedin P. (2014). Postpartum breast involution reveals regression of secretory lobules mediated by tissue-remodeling. Breast Cancer Res..

[100] Lund L.R., Rømer J., Thomasset N., Solberg H., Pyke C., Bissell M.J., Danø K., Werb Z. (1996). Two distinct phases of apoptosis in mammary gland involution: Proteinase-independent and -dependent pathways. Development.

[101] Kritikou E.A., Sharkey A., Abell K., Came P.J., Anderson E., Clarkson R.W., Watson C.J. (2003). A dual, non-redundant, role for LIF as a regulator of development and STAT3-mediated cell death in mammary gland. Development.

[102] Schere-Levy C., Buggiano V., Quaglino A., Gattelli A., Cirio M.C., Piazzon I., Vanzulli S., Kordon E.C. (2003). Leukemia inhibitory factor induces apoptosis of the mammary epithelial cells and participates in mouse mammary gland involution. Exp. Cell Res..

[103] Zhao L., Melenhorst J.J., Hennighausen L. (2002). Loss of Interleukin 6 Results in Delayed Mammary Gland Involution: A Possible Role for Mitogen-Activated Protein Kinase and Not Signal Transducer and Activator of Transcription 3. Mol. Endocrinol..

[104] Tiffen P.G., Omidvar N., Marquez-Almuina N., Croston D., Watson C.J., Clarkson R.W.E. (2008). A dual role for oncostatin M signaling in the differentiation and death of mammary epithelial cells in vivo. Mol. Endocrinol..

[105] Wyszynski A., Hong C.C., Lam K., Michailidou K., Lytle C., Yao S., Zhang Y., Bolla M.K., Wang Q., Dennis J. (2016). An intergenic risk locus containing an enhancer deletion in 2q35 modulates breast cancer risk by deregulating IGFBP5 expression. Hum. Mol. Genet..

[106] Takabatake Y., Oxvig C., Nagi C., Adelson K., Jaffer S., Schmidt H., Keely P.J., Eliceiri K.W., Mandeli J., Germain D. (2016). Lactation opposes pappalysin-1-driven pregnancy-associated breast cancer. EMBO Mol. Med..

[107] Basree M.M., Shinde N., Koivisto C., Cuitino M., Kladney R., Zhang J., Stephens J., Palettas M., Zhang A., Kim H.K. (2019). Abrupt involution induces inflammation, estrogenic signaling, and hyperplasia linking lack of breastfeeding with increased risk of breast cancer. Breast Cancer Res..

[108] Borges V.F., Lyons T.R., Germain D., Schedin P. (2020). Postpartum Involution and Cancer: An Opportunity for Targeted Breast Cancer Prevention and Treatments?. Cancer Res..

[109] Wambach K.A. (2003). Lactation mastitis: A descriptive study of the experience experiencia. J. Hum. Lact..

[110] Winder C.B., Sargeant J.M., Hu D., Wang C., Kelton D.F., Godkin M.A., Churchill K.J., O’Connor A.M. (2019). Comparative efficacy of antimicrobials for treatment of clinical mastitis in lactating dairy cattle: A systematic review and network meta-analysis. Anim. Health Res. Rev..

[111] Wu Y., Sun Y., Dong X., Chen J., Wang Z., Chen J., Dong G. (2020). The synergism of PGN, LTA and LPS in inducing transcriptome changes, inflammatory responses and a decrease in lactation aswell as the associated epigenetic mechanisms in bovine mammary epithelial cells. Toxins.

[112] Vangroenweghe F., Lamote I., Burvenich C. (2005). Physiology of the periparturient period and its relation to severity of clinical mastitis. Domest. Anim. Endocrinol..

[113] Taponen S., Pyorala S. (2009). Coagulase-negative staphylococci as cause of bovine mastitis—Not so different from *Staphylococcus aureus*?. Vet. Microbiol..

[114] Günther J., Esch K., Poschadel N., Petzl W., Zerbe H., Mitterhuemer S., Blum H., Seyfert H.M. (2011). Comparative kinetics of *Escherichia coli*-And *Staphylococcus aureus*-Specific activation of key immune pathways in mammary epithelial cells demonstrates that *S*. aureus elicits a delayed response dominated by interleukin-6 (IL-6) but not by IL-1A or tumor necrosis factor alpha. Infect. Immun..

[115] Barbosa-Cesnik C. (2003). Lactation Mastitis. JAMA.

[116] Sakemi Y., Tamura Y., Hagiwara K. (2011). Interleukin-6 in quarter milk as a further prediction marker for bovine subclinical mastitis. J. Dairy Res..

[117] Zhang Y., Wang X., Jiang Q., Hao H., Ju Z., Yang C., Sun Y., Wang C., Zhong J., Huang J. (2018). DNA methylation rather than single nucleotide polymorphisms regulates the production of an aberrant splice variant of IL6R in mastitic cows. Cell Stress Chaperones.

[118] Zhu Y., Berg M., Fossum C., Magnusson U. (2007). Proinflammatory cytokine mRNA expression in mammary tissue of sows following intramammary inoculation with *Escherichia coli*. Vet. Immunol. Immunopathol..

[119] Mizuno K., Hatsuno M., Aikawa K., Takeichi H., Himi T., Kaneko A., Kodaira K., Takahashi H., Itabashi K. (2012). Mastitis is associated with IL-6 levels and milk fat globule size in breast milk. J. Hum. Lact..

[120] Liu Y., Zhang J., Zhou Y.H., Jiang Y.N., Zhang W., Tang X.J., Ren Y., Han S.P., Liu P.J., Xu J. (2015). IL-6/STAT3 signaling pathway is activated in plasma cell mastitis. Int. J. Clin. Exp. Pathol..

[121] Liu Y., Sun Y., Zhou Y., Tang X., Wang K., Ren Y., He J. (2020). Sinomenine hydrochloride inhibits the progression of plasma cell mastitis by regulating IL-6/JAK2/STAT3 pathway. Int. Immunopharmacol..

[122] Schmid P., Adams S., Rugo H.S., Schneeweiss A., Barrios C.H., Iwata H., Diéras V., Hegg R., Im S.A., Shaw Wright G. (2018). Atezolizumab and Nab-Paclitaxel in Advanced Triple-Negative Breast Cancer. N. Engl. J. Med..

[123] Zhang W., Liu Y., Yan Z., Yang H., Sun W., Yao Y., Chen Y., Jiang R. (2020). IL-6 promotes PD-L1 expression in monocytes and macrophages by decreasing protein tyrosine phosphatase receptor type O expression in human hepatocellular carcinoma. J. ImmunoTher. Cancer.

